# Controlling forever love

**DOI:** 10.1371/journal.pone.0260529

**Published:** 2021-12-29

**Authors:** Jorge Herrera de la Cruz, José-Manuel Rey

**Affiliations:** 1 Department of Applied Mathematics and Statistics, CEU University, Madrid, Spain; 2 Department of Economic Analysis, Complutense University of Madrid, Madrid, Spain; Shandong University of Science and Technology, CHINA

## Abstract

A stable and rewarding love relationship is considered a key ingredient for happiness in Western culture. Building a successful long-term relationship can be viewed as a control engineering problem, where the control variable is the effort to be made to keep the relationship alive and well. We introduce a new mathematical model for the effort control problem of a couple in love who wants to stay together forever. The problem can be naturally formulated as a dynamic game in continuous time with nonlinearities. Adopting a dynamic programming approach, a tractable computational formulation of the problem is proposed together with an accompanying algorithm to find numerical solutions of the couple’s effort problem. The computational analysis of the model is used to explore feeling trajectories, effort control paths, happiness, and stabilization mechanisms for different types of successful couples. In particular, the simulation analysis provides insight into the pattern of change of both marital quality and effort making in intact marriages and how they are affected by certain level of heterogamy in the couple.

## Introduction

This article is concerned with the scientific study of long-term dyadic sentimental relationships. This is a theme of paramount importance in the social sciences since marriage is considered a “cultural universal” [1] and the fundamental social institution of most societies in world history [2, 3]. Also, marriage has significant implications on health and well-being. It has been consistently documented that high quality social relationships –and, notably, a functional marriage– have protective effects on the mental and emotional health of individuals [4]. Furthermore, there is a strong association between marital quality and overall happiness [5, 6]. Remarkably, the impact of marital quality on overall happiness has increased during the last decades [7], which reveals how important the role of a happy and stable union is nowadays in the quest for happiness. Probably in the pursuit of happiness, more than 90 percent of American people have married by age 50 [8].

Despite decades of research, it remains unclear what conditions are sufficient to build a long-term successful relationship. This is probably related to the fact that most studies have been typically focused on unsuccessful unions, due to the prevalence of divorce in Western societies [9]. Indeed, it is usually reported that about 50 percent of married couples, in the West, divorce [8]. Little is yet known about how trajectories differ between couples who break up and those who remain intact, which is partly due to the difficulty of monitoring relationships for a long time. In fact, long-term longitudinal marital studies are scarce and with limitations [10].

Given the deficit of longitudinal data, an alternative plan is to approximate long-term successful marital trajectories using computer simulation, which is our approach in this paper. We introduce a computer algorithm to generate trajectories for synthetic relationships characterized by well-founded conditions. The main advantage of this strategy is that the time frame can be extended as long as necessary, which is key to understanding long-lasting relationships.

Our computer simulation-based study stems from a new mathematical model for the dynamics of a romantic relationship. The state (quality) of the relationship is monitored by a time variable *x*(*t*) –called the *feeling* here– whose evolution is controlled by the couple through the effort each partner puts into the relationship, summarized by two variables, *c*_1_(*t*) and *c*_2_(*t*) –called the *effort* variables.

In terms of Sternberg’s triangular theory of love [11], *x*(*t*) can be thought to be related with the (non-rational) dimensions of love, i.e. passion and intimacy, while *c*_1_(*t*) and *c*_2_(*t*) may represent the (rational) dimension decision/commitment.

Dynamical systems theory provides a suitable toolbox for modeling the temporal evolution of a dyadic system, such as a romantic relationship. This mathematical approach was used in [12] to describe marital interaction in the laboratory and to repair negative trend dynamics. Also, dynamical systems were used in [13] to explain the initial stage of romantic relationships –what is called the *limerence syndrome* of love [14]. The same approach was used in [15] to analyze the intriguing problem of the complex sentimental interaction in a trio. The idea that the evolution of a relationship can be controlled via a dynamical system was first formalized in [16]. While [12] and [13] concern the interaction dynamics of the couple in the short or medium term, the model in [16] refers to the long-term evolution of the relationship, which is the relevant topic for the present study. This problem is linked to the *attachment syndrome* of love –see [14]. An accessible review of the mathematical models of love dynamics is provided in [17].

In most contemporary societies the prevailing formula for a happy lifelong relationship –typically marriage– is a version of Adam and Eve’s relationship. That means a monogamous union based on love, in which each partner considers the other the highest priority in life and expects most of his/her needs to be met within the pair [2, 18]. Building such a relationship is a very demanding task that requires great dedication [3, 9, 18].

It has long been recognized that love is a *conditio sine qua non* which is far from being sufficient for achieving success in a contemporary marriage [19]. Marital research, however, has provided consistent evidence of certain preconditions for thriving through a happy marriage. Firstly, commitment to the relationship seems an indispensable requirement: partners must engage themselves to build the relationship as a common project [9, 20, 21]. This is a basic ingredient in our modeling design.

Also, it seems a mistake to work on the relationship on the basis of reciprocity [21]. In our modeling we do not consider any interaction among the effort contributions of both partners, rather they enter additively into the equation for the feeling dynamics.

It is well-known that a significant level of similarity between partners –called homogamy– is associated with marital stability: homogamous couples are more likely to last –see e.g. [20, 22]. Homogamy is the usual form of pairing in the Western world, in accordance with the similarity-attraction paradigm [23]. Therefore, in our modeling design, we assume that couples are essentially homogamous. However, certain dissimilarities between partners may condition the development of the relationship –being gender the most obvious [24]. So we consider the possibility that partners differ in the efficiency of their effort contributions, and we analyze the impact of that heterogamous trait on the relationship.

At the core of our model formulation are the effort trajectories *c*_1_(*t*) and *c*_2_(*t*). An essential precondition for success is sufficient dedication to the relationship [19, 25]. A number of authors recommend different practices or strategies to build a successful relationship –see e.g. [18–21, 26]. All of them are rational activities that require making some effort. Our scheme is particularly aligned with the all-or-nothing marriage theory [18]. According to [18], “building and sustaining a high-quality marriage requires a deliberate effort”, and then the effort plan is formulated as “how much effort it would take”. This question amounts to determine the effort trajectories *c*_1_(*t*) and *c*_2_(*t*) for a successful relationship.

As suggested in [26], reason must be employed for controlling romantic love. This provides a rational criterion for raising the question of effort: the effort paths are determined for the couple to obtain maximal happiness. This is the couple’s effort problem that we address in this document.

In particular, we obtain a computational version of the problem together with an accompanying algorithm to generate trajectories for the synthetic couples of the model. The mathematical formulation of the effort control problem as a dynamic game along with the computational scheme of the model is presented in detail in the next section.

We exploit that our scheme allows us to compute trajectories for an arbitrarily long time to explore feeling and effort patterns of successful relationships, both homogamous and heterogamous. A significant contribution of our approach regards the pattern of decline of marital satisfaction in the long term, which is a matter of debate in the research literature –see [10]. Marital research so far has not provided evidence whether typical trajectories eventually decline, increase, or flatten out. Essentially, there are two perspectives to describe the trajectory of a successful relationship: after a continuous decline, either stabilization of marital quality follows (stability perspective) or a small upturn of marital quality occurs (resilience perspective). A recent study [10] gives support to the U-shaped pattern that is discarded by other authors –see e.g. [27]. It is certainly plausible that not all successful trajectories follow the same pattern. Our results suggest that both the stability and the resilience perspectives can occur for successful trajectories. While successful relationships with no exogenous stress show the stabilization pattern, those undergoing some episode of stress may exhibit a U-shaped feeling curve. Regarding the effort question, we find evidence that effort trajectories must increase until reaching a plateau as the feeling stabilizes. We also analyze resilience in heterogamous couples undergoing episodes of stress. We present and discuss our results below, in the Results and Discussion section. In the final section, we summarize our main findings for navigating a long and happy romantic relationship.

## Methods

In this section we introduce the mathematical formalization of the couple’s effort control problem, and its computational version to generate numerical solutions. We first present some general ideas about the problem statement and its mathematical treatment.

The effort control problem was interpreted as an infinite time horizon optimal control problem in [16], where the optimal effort path is determined so that total happiness is maximal. A similar model assuming a more general form of the feeling dynamics was analyzed in [28]. A mean-field stochastic version of this model was considered in [29]. This formulation considers the couple as a unit so that both feeling and effort are described by one-dimensional variables, and one common happiness integral is considered. This model fits well the case of homogamous couples made up of individuals similar to each other. To account for heterogamous couples, it must be assumed that partners can make efforts differently and therefore have a different happiness payoff. Mathematically, this involves modeling the effort problem as a dynamic two-person game in continuous time.

Considering a two-dimensional variable accounting for the feelings of each partner would provide a more complete description of the emotional state of the couple.

This model specification makes sense to account for couple interaction either to analyze the initial or transitional stages of the relationship –as in [13]– or to diagnose issues of influence and response between the partners –as in [12]. The interaction dynamics, however, is not so relevant for couples that are committed to sustain a long-term relationship after a successful period of adaptation, which is the problem considered in this paper.

In contrast to the optimal control analysis of the one-dimensional model in [16], we adopt here a dynamic programming approach to analyze dynamic games –see e.g. [30].

By solving a coupled system of Hamilton-Jacobi-Bellman (HJB) equations, effort feedback strategies are obtained that constitute a feedback Nash equilibrium of the dynamic love game. The value functions of the problem give the corresponding well-being of both members of the couple for the initial feeling.

A HJB formulation of the one-dimensional couple’s problem is considered in [31], where it is used to approximate numerically optimal open-loop effort solutions (i.e. effort is given as a function of time). However, feedback solutions (i.e. effort is given as a function of feeling) are not considered in [31]. Feedback effort solutions and value (well-being) functions both provide valuable information for the couple’s effort problem. In particular, feedback solutions allow partners to react to exogenous perturbations of the successful trajectory of the relationship. This is particularly useful to stabilize trajectories experiencing fluctuations. The dynamic programming approach adopted here supplements the open-loop analysis of the one-dimensional problem in [16].

The mathematical analysis of the dyadic model is challenging due to the functional structure of the optimization problems. In particular, the HJB equations for the dynamic love games must be solved numerically. A novel algorithm is proposed to find numerical solutions of the feedback Nash equilibria of the love game. The algorithm implemented here is based on the RaBVitG scheme introduced in [32] (see also [33]).

Our computational model finds feedback Nash equilibria for the couple’s effort problem. Nash equilibrium is the fundamental non-cooperative solution to social interaction problems [34]. In a Nash equilibrium of the differential love game, each partner determines his/her best effort path, maximizing his/her own happiness, knowing that the other partner does the same. This is not necessarily the cooperative solution in which both partners determine their common effort by maximizing their aggregate happiness. In general, real couples seem to have serious difficulties implementing cooperative solutions [35]. Still, there is an essential cooperative element in our love game model, as both partners are committed to keeping the relationship alive together by controlling the feeling dynamics. On the other hand, the fact that in the model each spouse seeks her happiness through the relationship complies with the contemporary vision of marriage [18].

### Differential love games

Let $x:\;[0,\infty )\to X\subseteq R^{+}$ be a real-valued differentiable function that describes the state of the relationship at every moment *t* ≥ 0. The variable *x*(*t*) essentially gives a measure of marital quality or relationship satisfaction at time *t* and it is called here the *feeling*.

The initial feeling *x*(0) = *x*_0_ is typically very large. According to [12], the feeling obeys the *second law of the thermodynamics for sentimental relationships*: there is a natural tendency of *x*(*t*) to decay in time, which can be counteracted with the effort made by both partners. The evolution law for the feeling variable can be written as the ordinary differential equation

$$\begin{array}{c} \frac{dx}{dt}\left(t\right)=-rx\left(t\right)+a_{1}c_{1}\left(t\right)+a_{2}c_{2}\left(t\right),\;t\geq 0, \end{array}$$ (1)

where *r*, *a*_1_, *a*_2_ > 0 and, for *i* = 1, 2, $c_{i}:\;[0,\infty )\to R^{+}$ is a (piecewise continuous) function measuring how much effort is made by partner *i* at every moment *t*. With no effort contribution, i.e. *c*_1_(*t*) = *c*_2_(*t*) = 0, Eq (1) implies that *x*(*t*) fades at a constant rate *r* > 0. The effort terms enter additively into the equation, following a recommended principle for successful relationships [21]. This linear decaying law is commonly accepted in the field and its adequacy is discussed in [16]. More general decaying laws are considered in [28]. The parameters *a*_1_, *a*_2_ > 0, given for each couple, measure the *efficiency* of a unit of effort of each partner. It must be understood that there is a threshold level *x*_min_ > 0 for the feeling below which the relationship is no longer viable.

Eq (1) is the state equation for the effort control problem of the couple. Each partner *i* seeks to determine the (optimal) effort path *c*_*i*_(*t*) in order to maximize his/her total well-being or happiness *W*_*i*_, which is defined by adding discounted instantaneous utilities over the (indefinite) life of the relationship, that is,

$$\begin{array}{c} W_{i}(c_{i})=\int_{0}^{\infty }e^{-\rho_{i}t}(U_{i}(x(t))-D_{i}\left(c_{1}(t),c_{2}(t)\right))dt,\;\;i=1,2. \end{array}$$ (2)

Here *ρ*_*i*_ > 0 is the individual rate of temporal preference, and *U*_*i*_ and *D*_*i*_ are (instantaneous) utility of feeling and disutility of effort, respectively. Both provide the well-being of partner *i* at every moment *t* in a cost-benefit fashion. Of course, the optimization problems of both partners are interdependent, since the control variables *c*_1_(*t*) and *c*_2_(*t*) both enter the state Eq (1) and (maybe) the happiness integrals *W*_1_ and *W*_2_, which makes the 2-person decision problem a game. Partner *i*’s functional is written above as a function of his/her control path *c*_*i*_(⋅) only, since that is his/her decision variable in his/her optimization problem.

The utility structure obeys the following specifications: *U*_*i*_ and *D*_*i*_ are (sufficiently) smooth functions such that, for *i* = 1, 2, $U_{i}^{\prime }\left(x\right)>0$, $U_{i}^{\prime \prime }\left(x\right)<0$, for *x* ≥ 0, and $U_{i}^{\prime }\left(x\right)\to 0$ as *x* → + ∞, whereas $\frac{\partial^{2}D_{i}}{\partial c_{i}^{2}}\left(c_{1},c_{2}\right)>0$, $\frac{\partial D_{1}}{\partial c_{1}}\left(c_{1}^{*},c_{2}\right)=0$ and $\frac{\partial D_{2}}{\partial c_{2}}\left(c_{1},c_{2}^{*}\right)=0$ for some $c_{1}^{*}\geq 0$, $c_{2}^{*}\geq 0$, and $\frac{\partial D_{i}}{\partial c_{i}}\left(c_{1},c_{2}\right)\to \infty$ as *c*_*i*_ → + ∞. Notice that, given *c*_*j*_ ≥ 0, the disutility *D*_*i*_ due to effort *c*_*i*_ reaches its absolute minimum at $c_{i}^{*}$, *i* = 1, 2. This cost-benefit utility scheme is based on well-known principles of human psychology. Essentially, the above assumptions imply that both the feeling *x* at any level and, for *i* = 1, 2, the effort *c*_*i*_, but only up to a certain level $c_{i}^{*}$, are a beneficial source of well-being at a decreasing rate, whereas the effort *c*_*i*_ beyond level $c_{i}^{*}$ is increasingly painful. The key parameter $c_{i}^{*}$ represents the level of effort preferred by partner *i*, *i* = 1, 2. In this article, it will be assumed that *D*_*i*_ depends on effort *c*_*i*_ only, that is, *D*_*i*_ = *D*_*i*_(*c*_*i*_) for *i* = 1, 2.

Now the *couple’s (effort) problem* can be stated as follows: given the feeling dynamics described by Eq (1), both partners determine effort trajectories *c*_1_(*t*), *c*_2_(*t*) that maximize their corresponding happiness integrals (2). This formulation corresponds to a two-person differential game with infinite time horizon [30].

In the particular case that *U*_1_ = *U*_2_, *D*_1_ = *D*_2_, *ρ*_1_ = *ρ*_2_, and *a*_1_ = *a*_2_, both partners are identical according to the structure of the problem. This type of couple is called *homogamous*, in accordance with the terminology in marital psychology (see, e.g. [36]). A variety of *heterogamous* couples can be considered by defining different inputs for each partner.

A solution of the couple’s problem is a pair $\left(c_{1}^{♡}\left(t\right),c_{2}^{♡}\left(t\right)\right)$ which solves the optimization problems of both partners simultaneously, given the state Eq (1) and the initial feeling *x*(0) = *x*_0_. This is called a Nash equilibrium of the differential game. Optimal solutions that lead to stationary (constant) feeling and effort values are of particular interest for the relationship, as they provide long-term happy and stable solutions with no fluctuations. Of course, a successful relationship requires that *x*(*t*) remains well above *x*_min_ for *t* ≥ 0 or at least most of the time.

A solution of a love differential game admits different representations, depending on the information available when both partners make their effort decision at time *t* ≥ 0. A control path *c*_*i*_(*t*) which only depends on *t* and the initial value *x*_0_ is called an *open-loop* solution or strategy. A partner *i* implementing an optimal open-loop solution observes the initial feeling *x*_0_ and commits to making effort *c*_*i*_(*t*) at time *t* ≥ 0, no matter what happens as time goes on. A representation of the optimal control path of the form *c*_*i*_(*t*) = *S*_*i*_(*x*(*t*), *t*) is called a closed-loop or *feedback* solution or strategy. A partner *i* implementing a feedback solution at time *t* ≥ 0 does need to observe the current state of the feeling *x*(*t*) and then makes effort *S*_*i*_(*x*(*t*), *t*). Open-loop solutions for differential games can be found, in principle, using control-theoretic methods.

A feedback approach is rather used in this paper to analyze the couple’s effort problem. Since it is plausible that *x*(*t*) can be observed by both partners when they make the effort decisions at time *t*, the feedback approach is particularly suitable here. Feedback strategies provide solutions for all effort control problems starting at time *t* > 0 with any given feeling level *x* [30]. They allow both partners to react to deviations of the feeling trajectory due to some external shock. This has significant implications for the success of a relationship in the long run. In the next section, several numerical experiments show how this idea is implemented for different relationships.

Since the time horizon is infinite and all input functions are time-independent, it makes sense to look for *stationary* feedback solutions, which are functions of the observed state *x* of the feeling only. In the dyadic case, a pair of strategies $\left(S_{1}^{♡}\left(\cdot \right),S_{2}^{♡}\left(\cdot \right)\right)$, $S_{i}^{♡}:\;X\to R^{+}$, is a *stationary feedback Nash equilibrium* of the differential game if $S_{i}^{♡}\left(x\left(t\right)\right)$ is an optimal effort control of partner *i*’s optimization problem. More specifically, $t\mapsto S_{1}^{♡}\left(x\left(t\right)\right)$ solves the problem

$$\underset{c_{1}\left(t\right)}{\text{max}}\int_{0}^{\infty }e^{-\rho_{1}t}(U_{1}(x(t))-D_{1}\left(c_{1}\left(t\right)\right)))dt,$$

with $\dot{x}\left(t\right)=-rx\left(t\right)+a_{1}c_{1}\left(t\right)+a_{2}S_{2}^{♡}\left(x\left(t\right)\right),\;x\left(0\right)=x_{0}$ and $c_{1}\left(t\right)\in R^{+}$, and $t\mapsto S_{2}^{♡}\left(x\left(t\right)\right)$ solves

$$\underset{c_{2}\left(t\right)}{\text{max}}\int_{0}^{\infty }e^{-\rho_{2}t}(U_{2}(x(t))-D_{2}\left(c_{2}\left(t\right)\right))dt,$$

with $\dot{x}\left(t\right)=-rx\left(t\right)+a_{1}S_{1}^{♡}\left(x\left(t\right)\right)+a_{2}c_{2}\left(t\right),\;x\left(0\right)=x_{0}$ and $c_{2}\left(t\right)\in R^{+}$. As already mentioned, we assume that *D*_*i*_ depends solely on effort *c*_*i*_, for *i* = 1, 2.

Assume that a stationary feedback Nash Equilibrium $S^{♡}=\left(S_{1}^{♡},S_{2}^{♡}\right)$ exists for the couple’s effort problem. Let $v_{i}^{♡}:\;X\to R$ be the value function of partner *i*, defined by

$$\begin{array}{c} v_{i}^{♡}(x_{0})=W_{i}(S_{i}^{♡}(x(t))),\;i=1,2, \end{array}$$ (3)

where $S_{i}^{♡}(x(t))$ is the feedback solution of the control problem of partner *i*, with initial state *x*(0) = *x*_0_. Under suitable conditions (see e.g. [37]), the value functions $v_{1}^{♡}\left(x\right)$ and $v_{2}^{♡}\left(x\right),\;x\in X$, are solutions of the following coupled system of Hamilton-Jacobi-Bellman (HJB) equations,

$$\begin{array}{c} \left\{ \begin{array}{c} \rho_{1}v_{1}\left(x\right)={\text{max}}_{c_{1}\in R^{+}}\{U_{1}(x)-D_{1}(c_{1})+v_{1}^{\prime }\left(x\right)(-rx+a_{1}c_{1}+a_{2}S_{2}\left(x\right))\},\\ \rho_{2}v_{2}\left(x\right)={\text{max}}_{c_{2}\in R^{+}}\{U_{2}(x)-D_{2}(c_{2})+v_{2}^{\prime }\left(x\right)(-rx+a_{1}S_{1}\left(x\right)+a_{2}c_{2})\}. \end{array}\right. \end{array}$$ (4)

Solving (4) (stationary) Nash equilibrium feedback solutions for each partner, $S_{i}^{♡}:\;X\to R^{+}$, *i* = 1, 2, are defined as

$$\begin{array}{c} \left\{ \begin{array}{c} S_{1}^{♡}\left(x\right)\in arg{\text{max}}_{c_{1}\in R^{+}}\{U_{1}(x)-D_{1}(c_{1})+v_{1}^{\prime }\left(x\right)(-rx+a_{1}c_{1}+a_{2}S_{2}^{♡}\left(x\right))\},\\ S_{2}^{♡}\left(x\right)\in arg{\text{max}}_{c_{2}\in R^{+}}\{U_{2}(x)-D_{2}(c_{2})+v_{2}^{\prime }\left(x\right)(-rx+a_{1}S_{1}^{♡}\left(x\right)+a_{2}c_{2})\}. \end{array}\right. \end{array}$$ (5)

In the case that $S_{i}^{♡}\left(x\right)$ is a singleton for all *x* ∈ *X*, *i* = 1, 2, the (equilibrium) feedback maps can be plugged into (1) to obtain

$$\begin{array}{c} \frac{dx}{dt}\left(t\right)=-rx\left(t\right)+a_{1}S_{1}^{♡}\left(x\left(t\right)\right)+a_{2}S_{2}^{♡}\left(x\left(t\right)\right), \end{array}$$ (6)

which, along with *x*(0) = *x*_0_, gives the optimal feeling evolution $x^{♡}\left(t\right)$ of the couple’s problem with initial feeling state *x*_0_. In what follows we call $c_{i}=S_{i}^{♡}\left(x\right)$ the *effort feedback* map of partner *i*, *i* = 1, 2.

The theoretical scheme for the analysis of the differential love games proposed in this paper can be summarized as follows. Assume that a couple’s effort problem has been defined by specifying the model inputs, namely utility and disutility functions *U*_1_, *U*_2_ and *D*_1_, *D*_2_, and parameters *r*, *ρ*_1_, *ρ*_2_, and *a*_1_, *a*_2_. Then, provided that the system of HJB Eq (4) can be solved and the optimization problems on the right-hand side of (4) have unique solutions, the basic outputs for the couple’s problem are the value (well-being) function $v_{i}^{♡}\left(x\right)$ and the effort feedback map $S_{i}^{♡}\left(x\right)$ of each partner *i* = 1, 2.

### A computational model of differential love games

General existence or uniqueness results for feedback Nash equilibria are not available in the literature [38]. Except for some particular cases that are analytically tractable, a computational approach is required to find solutions of differential games with nonlinearities, like the love games defined in this paper. Still, computational differential games is a young field with a very limited number of numerical methods available –see [39].

In this section, we introduce a computational model for the couple’s effort problem. Our goal is to generate efficient numerical approximations of the well-being functions $v_{i}^{♡}\left(x\right)$ and effort feedback maps $S_{i}^{♡}\left(x\right),\;i=1,2,$ defined by a differential love game. This entails designing an algorithm to solve the HJB dyadic system (4). First, a Semi-Lagrangian approximate version of (4) is obtained by time and space discretization, as in [40] or [41]. Then a mesh-free numerical scheme, that uses radial basis functions approximation and a double loop of value and game iteration, adapted from [32], is implemented to solve the discretized HJB system.

Let *h* > 0 be a small time step, and *t*_*k*_ = *kh*, k∈N∪{0}.

Given a state value *y* ∈ *X*, consider the following discrete version of (2):

$$\begin{array}{c} W_{i}^{h}(c_{i}^{h})=h\sum_{k=0}^{\infty }e^{-\rho_{i}t_{k}}(U_{i}(x_{k})-D_{i}(c_{i,k})),\;i=1,2, \end{array}$$ (7)

where $c_{i}^{h}={\left\{c_{i,k}\right\}}_{k\in N\cup \{0\}}$ is a sequence of feasible controls of partner *i*, which defines the piecewise constant function $c_{i}^{h}\left(\tau \right)=c_{i,k},\;\tau \in [t_{k},t_{k+1})$. The sequence *x*_*k*_ = *x*(*t*_*k*_) is obtained by a first-order Euler scheme for Eq (1), that is

$$\begin{array}{c} x_{k+1}=x_{k}+hf\left(x_{k},c_{1,k},c_{2,k}\right),\;x_{0}=y,\;k\geq 0, \end{array}$$ (8)

where *f* denotes the feeling dynamics *f*(*x*, *c*_1_, *c*_2_) = −*rx* + *a*_1_ *c*_1_ + *a*_2_ *c*_2_. A discrete version of (3) is thus given by

$$\begin{array}{c} v_{i}^{h}\left(y\right)=\underset{c_{i}^{h}}{\text{max}}\;W_{i}^{h}(c_{i}^{h}). \end{array}$$ (9)

From now on, the superindex in $S_{i}^{♡}\left(\cdot \right)$ and $v_{i}^{♡}\left(\cdot \right)$ is dropped to simplify notation. Following [41], a first order discrete-time HJB version of (4) is obtained as

$$\begin{array}{c} \left\{ \begin{array}{cc} v_{1}^{h}\left(y\right)={\text{max}}_{c_{1}\in R^{+}}\{h(U_{1}(y)-D_{1}(c_{1}))+(1-\rho_{1}h)v{}_{1}^{h}(y+hf(y,c_{1},S_{2}^{h}\left(y\right)))\}, & \;\\ v_{2}^{h}\left(y\right)={\text{max}}_{c_{2}\in R^{+}}\{h(U_{2}(y)-D_{2}(c_{2}))+(1-\rho_{2}h)v{}_{2}^{h}(y+hf(y,S_{1}^{h}\left(y\right),c_{2}))\}, \end{array}\right. \end{array}$$ (10)

where the corresponding discrete versions of (5) are defined as

$$\begin{array}{c} \left\{ \begin{array}{cc} S_{1}^{h}\left(y\right)\in arg{\text{max}}_{c_{1}\in R^{+}}\{h(U_{1}(y)-D_{1}(c_{1}))+(1-\rho_{1}h)v{}_{1}^{h}(y+hf(y,c_{1},S_{2}^{h}\left(y\right)))\}, & \;\\ S_{2}^{h}\left(y\right)\in arg{\text{max}}_{c_{2}\in R^{+}}\{h(U_{2}(y)-D_{2}(c_{2}))+(1-\rho_{2}h)v{}_{2}^{h}(y+hf(y,S_{1}^{h}\left(y\right),c_{2}))\}. \end{array}\right. \end{array}$$ (11)

Numerical approximations of the functions $v_{i}^{h}$ satisfying (10) are obtained via spatial discretization of the state space as follows. Let $\tilde{X}={\left\{y_{j}\right\}}_{j=1,\ldots ,Q}\subset X$ be a set of *Q* arbitrary points. Notice that the values $y_{i}^{♯}=y_{j}+hf(y_{j},c_{1},c_{2})$ appearing in (10) are not necessarily in $\tilde{X}$. To find approximate values ${\tilde{v}}_{i}^{h}(y_{j})$ of $v_{i}^{h}(y_{j})$ for $y_{j}\in \tilde{X}$, *i* = 1, 2, the values $v_{i}^{h}\left(y^{♯}\right)$ in (10) are computed by a collocation algorithm using a mesh-free method based on scattered nodes –see [42]. More precisely, the HJB scheme (10) and (11) is approximated on the nodes $y_{j}\in \tilde{X},\;j=1,\ldots ,Q$, by

$$\begin{array}{c} \left\{ \begin{array}{c} {\tilde{v}}_{1}(y_{j})={\text{max}}_{c_{1}\in R^{+}}\{h(U_{1}(y_{j})-D_{1}(c_{1}))+(1-\rho_{1}h)RBF[V_{1}](y_{1}^{\#})\},\\ {\tilde{v}}_{2}(y_{j})={\text{max}}_{c_{2}\in R^{+}}\{h(U_{2}(y_{j})-D_{2}(c_{2}))+(1-\rho_{2}h)RBF[V_{2}](y_{2}^{\#})\}, \end{array}\right. \end{array}$$ (12)

along with the corresponding discretized feedback strategies

$$\begin{array}{c} \left\{ \begin{array}{c} {\tilde{S_{1}}}^{h}\left(y_{j}\right)\in arg\;{\text{max}}_{c_{1}\in R^{+}}\{h(U_{1}(y_{j})-D_{1}(c_{1}))+(1-\rho_{1}h)RBF[V_{1}](y_{1}^{\#})\},\\ {\tilde{S_{2}}}^{h}\left(y_{j}\right)\in arg\;{\text{max}}_{c_{2}\in R^{+}}\{h(U_{2}(y_{j})-D_{2}(c_{2}))+(1-\rho_{2}h)RBF[V_{2}](y_{2}^{\#})\}, \end{array}\right. \end{array}$$ (13)

being

$$\left\{ \begin{array}{c} y_{1}^{\#}=y_{j}+hf(y_{j},c_{1},{\tilde{S_{2}}}^{h}\left(y_{j}\right)),\\ y_{2}^{\#}=y_{j}+hf(y_{j},{\tilde{S_{1}}}^{h}\left(y_{j}\right),c_{2}), \end{array}\right.$$

where *RBF*[*V*_*i*_](⋅) above denotes the approximation of ${\tilde{v}}_{i}^{h}(y_{i}^{♯})$ using the method of Radial Basis Functions [43]. The algorithm to implement the computational model is designed using the RaBVitG structure introduced in [32].

The details of the RaBVitG scheme, together with a computational analysis of its efficiency and accuracy can be found in [33]. A pseudocode of the algorithm used in this paper is provided in the S1 File.

## Results and discussion

Recall that the basic inputs for the numerical analysis are: the feeling erosion parameter *r* > 0 plus the effort efficiency coefficients *a*_1_, *a*_2_ > 0, which characterize the feeling dynamics (1), and the utility structure of both partners, given by the functions *U*_1_, *U*_2_ and *D*_1_, *D*_2_, plus the discount factors *ρ*_1_, *ρ*_2_ > 0, which define the happiness integrals in (2). The main outputs of the algorithm, namely the (optimal) effort feedback maps *S*_1_(⋅), *S*_2_(⋅) and the well-being functions *v*_1_(⋅), *v*_2_(⋅) are obtained from (12) and (13), respectively. In the one-dimensional (1D) case, a feedback rule *S*(⋅) and a value function *v*(⋅) are estimated for the couple, considered a unit. Given the initial feeling *x*_0_, in the two-dimensional (2D) problem the evolution of a successful relationship is obtained from the numerical scheme

$$\left(\text{SM}1\right)\;\;\{\begin{array}{c} c_{i,k}={\tilde{S_{i}}}^{h}\left(x_{k}\right),\;i=1,2,\\ x_{k+1}=x_{k}+hf\left(x_{k},c_{1,k},c_{2,k}\right),\;\;k\geq 0,\\ x_{0}\in X, \end{array}$$

where *h* > 0 is the time step of the computational model and the discretized feedback maps ${\tilde{S}}_{i}^{h}$, *i* = 1, 2, are defined by (13). As already mentioned, ${\tilde{S}}_{i}^{h}$ is defined by RBF approximation for values different from the nodes $y_{j}\in \tilde{X}$.

Only one effort variable *c*_*k*_ appears in the 1D version of the scheme.

Feedback strategies allow partners to react optimally to a perturbation of the feeling state at any time. If a shock *σ*_*k*_ alters the feeling trajectory with initial state *x*_0_ at a certain period *k* > 0, partners can compute the new optimal solution taking the altered feeling state as the starting condition and using their effort feedback maps accordingly. This *stabilization mechanism* is implemented in the 2D case by the scheme

$$\left(\text{SM}2\right)\;\;\{\begin{array}{c} c_{i,k}^{\sigma }={\tilde{S}}_{i}^{h}\left(x_{k}^{\sigma }\right),\;i=1,2,\\ x_{k+1}^{\sigma }=x_{k}^{\sigma }+hf\left(x_{k}^{\sigma },c_{1,k}^{\sigma },c_{2,k}^{\sigma }\right)+\sigma_{k+1},\;\;k\geq 0,\\ x_{0}^{\sigma }\in X, \end{array}$$

where *σ* = {*σ*_*k*_}_*k*_ is a sequence of punctual shocks. Typically, shocks are not persistent in real situations, so *σ* will be an array of zeroes except for a few elements (see e.g. Fig 2 below). Again, values of ${\tilde{S}}_{i}^{h}$ for arbitrary state values are obtained by RBF approximation. In the 1D case, only one control variable appears in the scheme (SM2).

Given *x*_0_, the control paths $c_{i,k}={\tilde{S_{i}}}^{h}\left(x_{k}\right)$ (*i* = 1, 2) obtained from (SM1) constitute an open-loop numerical solution of the couple’s effort problem. The scheme (SM2) allows us to compare the stabilizing solution with the unperturbed solution.

Two versions of the couple’s effort problem are considered next. First, the 1D model of the problem is analysed. The feedback approach provides here valuable complementary information to the (open-loop) control-theoretic methods employed in [16]. Then, the analysis of the dyadic (2D) version of the problem is addressed.

The same parameter values are used in both numerical studies. They can be seen in Table 1. The utility and disutility functions used for the analysis, namely

$$\begin{array}{c} U_{i}\left(x\right)=5\;\text{ln}\left(x+1\right),\;D_{i}\left(c_{i}\right)=\frac{1}{2}{\left(c_{i}-c_{i}^{*}\right)}^{2},\;\;i=1,2, \end{array}$$ (14)

are the same as those considered in [31]. This choice is useful to extend their open-loop analysis of the problem. The utility and disutility functions above satisfy the model specifications required in the previous section. The numerical results presented in this section are robust with respect to different specifications of the model inputs.

**Table 1:** Parameter values and utility/disutility functions.

	*i*	*r*	*ρ*_*i*_	*U*_*i*_	*D*_*i*_	$c_{i}^{*}$
1D model	1	−2	0.1	5 ln (*x* + 1)	$\frac{\left(c_{i}-c_{i}^{*}\right)}{2}$	0.2
2D model	1,2					

The table shows the numerical and functional inputs for the computational experiments presented in the Results and Discussion section. Functional forms are the same as those used in [31].

### 1D couple’s problem: Feedback analysis, lovebirds trajectories, and stabilization

The 1D version of the couple’s effort problem was considered in [31], who proved the existence of a unique solution for the “lovebirds problem”, that is, given an initial feeling *x*(0) = *x*_0_, there is a unique solution *c*^♡^ (*t*) for the couple’s effort problem.

Furthermore, for any initial feeling *x*_0_, the corresponding optimal trajectory (*c*^♡^ (*t*), *x*^♡^ (*t*)) converges towards the unique equilibrium $\left({\bar{c}}^{♡},{\bar{x}}^{♡}\right)$ of the following dynamical system, which is obtained from Pontryagin’s maximum principle,

$$\begin{array}{c} \left\{ \begin{array}{c} \frac{dx}{dt}\left(t\right)=-rx\left(t\right)+ac\left(t\right),\\ \frac{dc}{dt}\left(t\right)=(D^{\prime \prime }\left(c\left(t\right)\right){)}^{-1}\{\left(r+\rho \right)D^{\prime }\left(c\left(t\right)\right)-aU\left(x\left(t\right)\right)\}. \end{array}\right. \end{array}$$ (15)

The equilibrium $\left({\bar{c}}^{♡},{\bar{x}}^{♡}\right)$ is a saddle point, so the optimal trajectory lies on the stable manifold of the system (see [16] or Theorem 1 in [31]).

**Fig 1:**
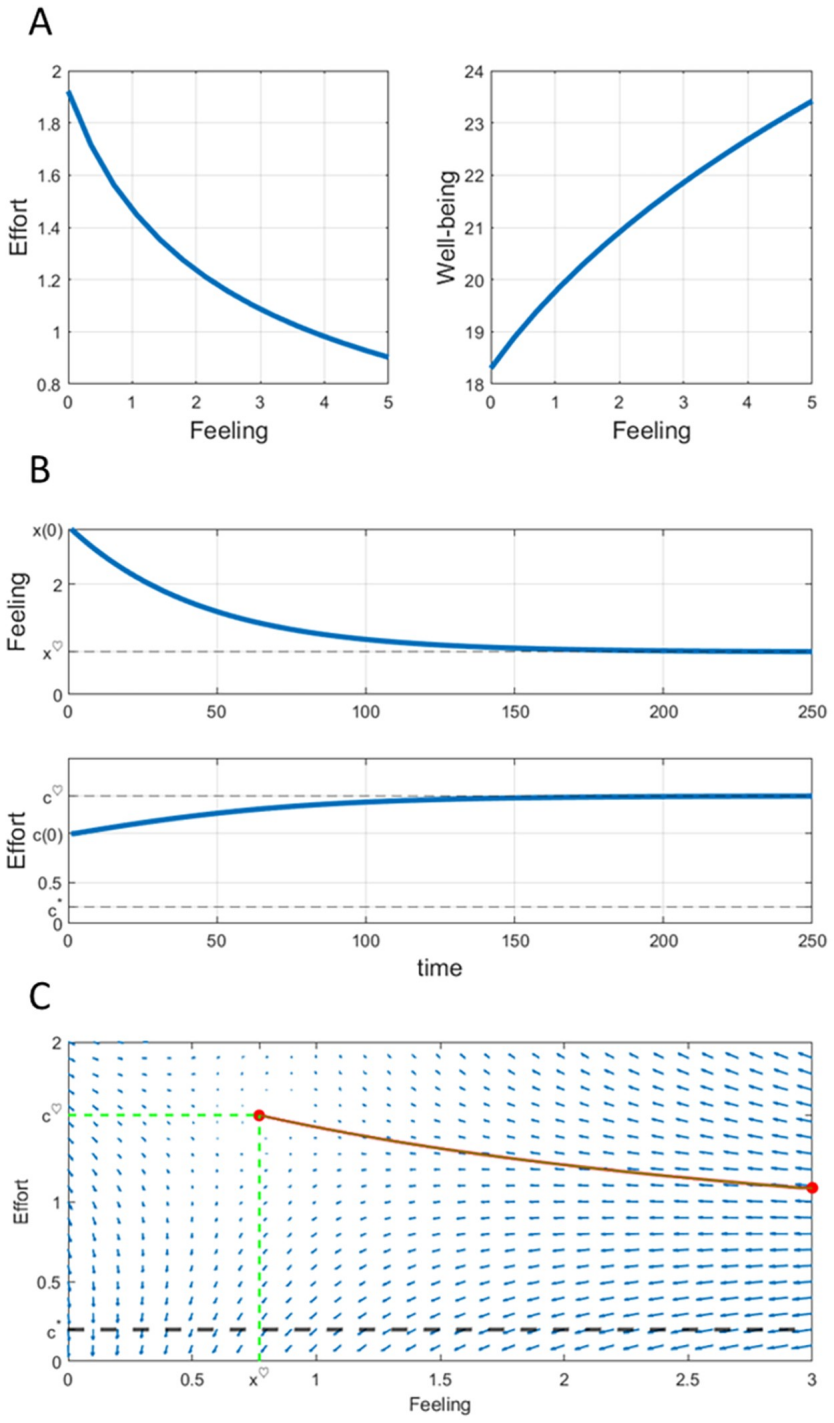
Computational feedback analysis for the one-dimensional couple’s effort problem. Numerical inputs for the analysis are given in Table 1. (A) represents the effort feedback map and well-being function, (B) shows the open-loop trajectories: feeling and effort time solutions for *x*(0) = 3 (same initial value as in [31]). For this initial value, the initial effort computed by the algorithm is *c*(0) ≃ 1.087. (C) shows the vector field of the effort-feeling system (15). The trajectory in red corresponds to the initial value *x*(0) = 3, for which *c*(0) = 1.087 is the initial required effort computed by the algorithm. The time evolution of this solution is shown in (B). This is the “lovebirds” trajectory for *x*(0) = 3, according to [31].

Let *a* = 1 for the subsequent analysis. The main outputs of the feedback analysis are shown in Fig 1. The effort feedback map together with the well-being function for feeling levels *x* ∈ [0, 5] are given in Fig 1A. The optimal effort policy depends on the feeling level in a decreasing fashion, whereas well–being increases with the initial feeling. These are general patterns that may be of some practical use to deal with the couple’s problem. The initial feeling matters for total happiness: successful couples that initially are deeply in love obtain more happiness from their relationship. They must be prepared, however, to increase their effort if the quality of the relationship declines regardless of its level.

Fig 1B shows the trajectories of optimal effort and feeling, computed using the feedback scheme (SM1) for the initial condition *x*(0) = 3. As time increases, the feeling decreases towards a stationary value, and the optimal effort also approaches an equilibrium value in an increasing manner. The analysis shows that the effort trajectory lies above the preferred effort level (c* = 0.2 in our simulation). So, sustaining a happy relationship requires an extra effort with respect to the favorite level c*, regardless of its value. This is a general result that was established in [16]. Therefore, successful couples must expect a continuous decline of relationship quality until a plateau is eventually reached. This pattern is in accord with the stability perspective of marriage –see [10]. Marriages that end in divorce are expected to show a steady and uninterrupted decline in marital quality until they finally break up. The typical trajectory obtained here for intact couples, however, shows an initial decay followed by stabilization.

Successful couples must increase their effort over time as marital quality declines, until both feeling and effort eventually approach constant levels. As mentioned above, a successful effort trajectory lies above the favorite level. Maintaining a rewarding relationship is always costly in terms of effort: no matter how much effort a partner is willing to put in, the required effort is more demanding.

Fig 1C shows the vector field of the feeling-effort dynamics (15) computed using a Runge-Kutta numerical scheme. It shows a particular example of the phase portrait of the 1D couple’s problem (see Fig 3 in [16]). The optimal trajectory (*c*^♡^ (*t*), *x*^♡^ (*t*)) for *x*_0_ = 3 computed by the algorithm is plotted in red. The trajectory lies on the stable manifold and approaches the equilibrium levels $\left({\bar{c}}^{♡},{\bar{x}}^{♡}\right)=\left(0.77,1.55\right)$ as *t* increases, in accordance with the computation in [31]. The red curve in Fig 1C shows the coupled evolution of the feeling and effort of a successful relationship: the required level of effort is higher than the preferred level c* = 0.2 and increases over time, while the feeling –initially high– declines. Eventually, both variables enter a stationary regime in which the feeling is sufficiently gratifying and the effort remains beyond the favorite level.

The feedback scheme (SM1) takes the computed value *c*^♡^ (0) as the initial condition to yield the open-loop solution for the “lovebirds problem” considered in [31]: given the initial feeling *x*(0), the algorithm finds the initial effort *c*(0) so that the whole trajectory (*c*^♡^ (*t*), *x*^♡^ (*t*)) lies on the stable manifold of (15). The feedback analysis of Fig 1A extends the open-loop numerical analysis of [31].

**Fig 2:**
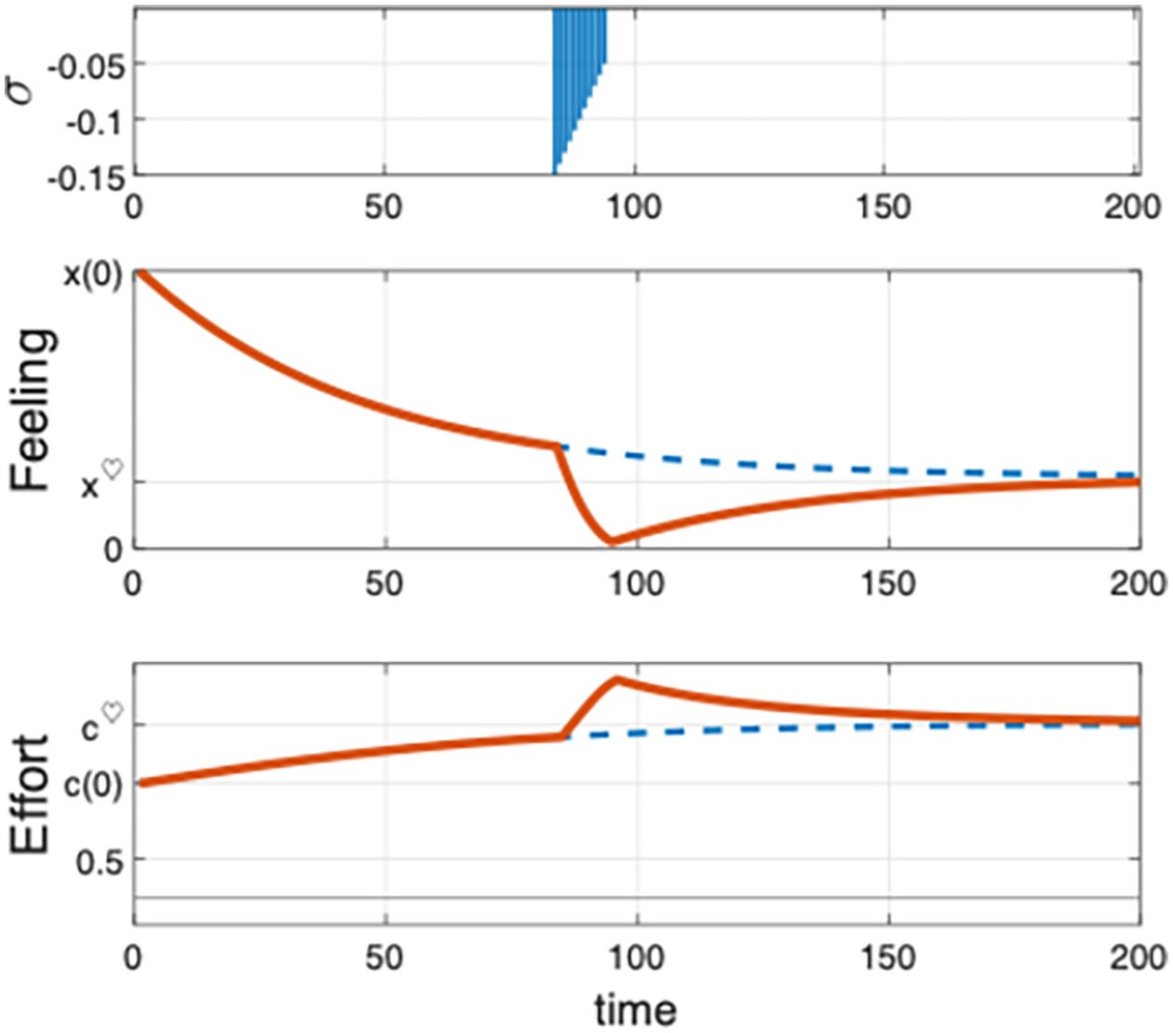
Feedback stabilization for the one-dimensional couple’s effort problem. The first panel shows the exogenous perturbation of the successful feeling trajectory. The sequence of shocks begins in the period *t* = 85 and takes values from −0.15 and −0.05, over 12 consecutive periods. The panels below show the corresponding feedback stabilizing solution (in red). The second panel shows the feeling trajectory along with the unperturbed solution (in blue). The third panel shows the stabilizing effort trajectory together with the unperturbed effort path (in blue). Initial feeling is *x*(0) = 3.

Fig 2 exhibits the stabilization mechanism for the couple’s problem. Open-loop effort and feeling solutions obtained from (SM1) for *x*(0) = 3 are shown in blue. For the particular sequence of perturbations shown in the first panel, the stabilizing solution is computed using the feedback scheme (SM2). The graphs of the corresponding effort and feeling are displayed in red in the figure, together with the unperturbed solutions (in blue). Assuming a month as the time period in our discrete model, the perturbation considered in Fig 2 corresponds to a sequence of twelve consecutive decreasing shocks taking place throughout the seventh year of the relationship. This stressful event is consistent with the popular “seven-year itch”. It has been argued that the risk of divorce follows a U-inverted pattern over time, with the peak around the seventh year of marriage [44, 45]. If the perturbation is too large or persistent, the feeling can approach low values and the survival of the relationship is at risk during the critical period (recall that *x*(*t*) should remain above a certain threshold value *x*_min_.

In our simulation –see Fig 2–, there is a pronounced decline of the feeling as a consequence of the one-year shock. The feeling reaches the lowest level after a year and then, only after a slow recovery, it comes close to pre-shock levels. Fig 2 also shows that, over the critical period, driving the feeling back to the successful trajectory requires extra effort, in particular, larger than the stationary level ${\bar{c}}^{♡}$. This is an additional source of stress to get back on the successful track.

Couples committed to thriving in the long term are probably aware that they will be facing certain stressful life events –some of them unpredictable. Our analysis suggests that they should be prepared for a noticeable impact on the quality of their relationship and go the extra mile for some time to bring the relationship (slowly) back to the pre-crisis level. This scenario of distress probably cannot be handled by many couples, and they eventually break up. The analysis may explain the burst in breakups over a specific stage of a relationship, like the seven-year itch. It also accounts for the inverted U-shaped curve of divorce risk. On the other hand, the feedback analysis shows that, for successful marriages overcoming stressful periods, marital quality exhibits a U-shape pattern over time, as it is shown in Fig 2. This result is consistent with the *marital resilience perspective* for marriages that avoid divorce –see [10].

### 2D couple’s problem: Feedback analysis and heterogamy effects

A dyadic modeling of the couple’s problem permits a richer analysis, accounting for the effect of asymmetry in the model inputs. In the case that *U*_1_ = *U*_2_, *D*_1_ = *D*_2_, *ρ*_1_ = *ρ*_2_, and *a*_1_ = *a*_2_, a couple is called homogamous, whereas if some model input differs, the couple is heterogamous. To illustrate how heterogamous and homogamous couples compare, all inputs are kept identical except for the parameters *a*_1_ and *a*_2_. They represent the efficiency of each partner’s effort. Assuming *a*_2_ ≠ *a*_1_ implies that the same level of effort impacts differently on the feeling in the short term whether it is made by one partner or the other. The efficiency parameter is a reasonable choice to analyze dissimilarity within the couple without undermining homogamy much, which is known to contribute to stability.

**Fig 3:**
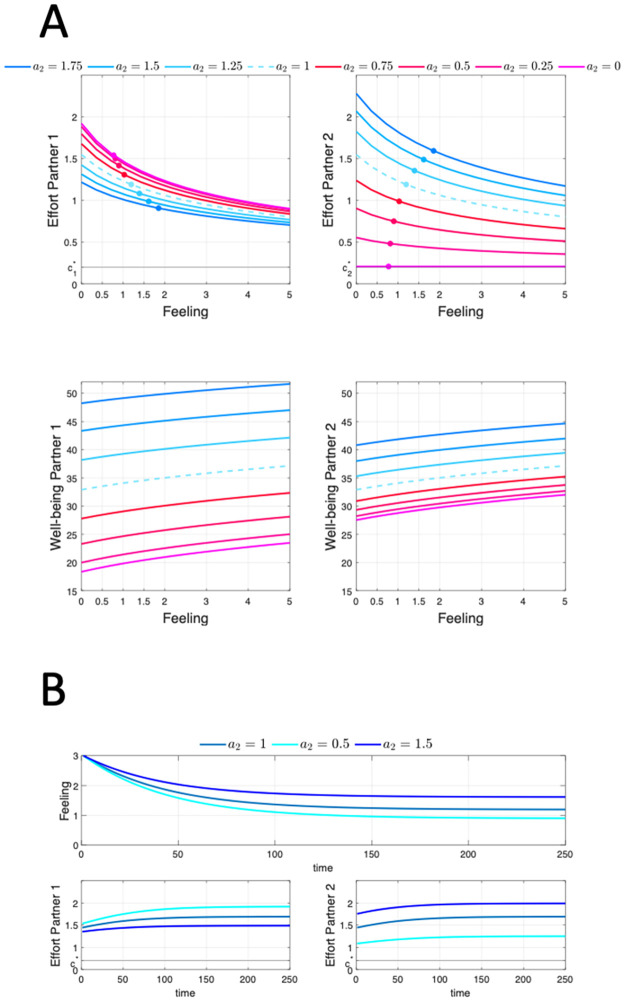
Computational feedback analysis of the two-dimensional couple’s effort problem. Numerical inputs for the analysis are given in Table 1. (A) shows the effort feedback maps and well-being functions for each partner. A set of curves in the same color corresponds to a type of heterogamous couple, with efficiency parameters *a*_1_ = 1 and *a*_2_ ∈ {0, 0.25, 0.5, 0.75, 1, 1.25, 1.5, 1.75}. The heterogamy gap *a*_2_ − *a*_1_ (*a*_1_ = 1) increases as color changes from purple to dark blue. The dashed blue curve corresponds to the homogamous case. The points marked on each curve correspond to the equilibrium levels of feeling and effort. (B) shows the feeling and effort trajectories for homogeneous and heterogeneous couples. Initial feeling is *x*(0) = 3.

The coefficient *a*_1_ = 1 will be fixed for the analysis, whereas *a*_2_ will be varied within the range [0, 1.75], which includes the homogamous case *a*_1_ = *a*_2_ = 1. The rest of input parameters and functions for the computation are as specified in Table 1. The feedback analysis for different pairs (*a*_1_ = 1, *a*_2_) is presented in Fig 3. The effort feedback maps and the corresponding value functions are shown in Fig 3A. Each set of four different curves in the same color corresponds with the output of the algorithm for a given pair (*a*_1_, *a*_2_), as specified in the key of the figure. The homogamous pair *a*_1_ = *a*_2_ = 1 corresponds to the dotted line, that can be considered the benchmark case. For any individual pair (*a*_1_, *a*_2_), the dyadic effort feedback maps and well-being functions show the same qualitative pattern as in the 1D model. In general, as the level of feeling decreases, the individual effort of both partners must increase. On the other hand, their happiness is higher as the initial feeling increases. The analysis reveals that, as *a*_2_ increases, the effort feedback curves of partner 1 monotonically decrease while those of partner 2 monotonically increase. Thus, as the effort efficiency of partner 2 increases, partner 1 lowers his/her effort while partner 2 increases his/hers, regardless of the level of feeling. Also, the partner who is more efficient puts more effort into the relationship. For any pair (*a*_1_ = 1, *a*_2_), all effort curves are above the favorite effort level ($c_{1}^{*}=c_{2}^{*}=0.2$). The simulation provides numerical evidence of the existence of an effort gap, namely for any feeling level, both partners must make an effort beyond their preferred level. The existence of an effort gap was established in [16] in the 1D formulation of the couple’s problem. In that regard, the case (*a*_1_ = 1, *a*_2_ = 0) seems relevant here: the effort curve of partner 2 is flat at the favorite level $c_{2}=c_{2}^{*},$ so when the effort made by partner 2 has no effect, his/her effort gap is null and the relationship survives at the cost of the effort gap of partner 1 being maximal. Regarding how heterogamy impacts happiness, the analysis shows that, for any initial feeling level, the well-being of both partners increases as the effort efficiency of partner 2 rises. The effect is more pronounced for partner 1. Indeed, when partner 2 is more (respectively, less) efficient than partner 1, the increase (respectively, decrease) in partner 1’s well-being is significantly larger than that in partner 2 –see Fig 3A. The case (*a*_1_ = 1, *a*_2_ = 0), in which the effort gap of partner 2 is null, entails the worst-case scenario for the well-being of both partners, regardless of the initial feeling. Also, in this case partner 1’s well-being is lower than partner 2’s for any initial feeling level.

Open-loop trajectories of feeling and effort show the same pattern of convergence towards stationarity as in the 1D model –see Fig 1B. Three different successful trajectories are represented in Fig 3B, namely for (*a*_1_ = 1, *a*_2_ = 1), (*a*_1_ = 1, *a*_2_ = 0.5), and (*a*_1_ = 1, *a*_2_ = 1.5). The initial feeling is the same in all cases. The feeling always decreases towards a stationary value which is always higher for the couple with a higher *a*_2_. Of course, the effort trajectories of the partners of the homogamous are identical. However, as anticipated by the effort feedback curves in Fig 3A, partners in a heterogamous couple must make different levels of effort. Their effort increases over time until eventually approaching their equilibrium level, which is higher for the most efficient partner. The disparity in effort comes at a cost to the most efficient partner, as he/she must endure a larger effort gap throughout the relationship. Our simulation suggests that partners in heterogamous couples must be willing to tolerate a different effort gap relative to their preferred level of effort. Fig 3B contains a useful suggestion for choosing a lifetime partner: choosing a partner who is more efficient than yourself will bring you more happiness, and it will also entail a lower effort investment on your part through the entire relationship.

Successful feeling and effort trajectories eventually approach stationary levels, and the relationship reaches an equilibrium.

Feeling and effort levels at equilibrium depend on the heterogamous character of the couple, as it can be seen in Fig 3A. The points in the same color lying on the effort curves of both partners correspond to the equilibrium level of each couple. As the heterogamy gap *a*_2_ − *a*_1_ increases, the feeling level increases, and the effort gap of partner 1 improves while the effort gap of partner 2 worsens.

**Fig 4:**
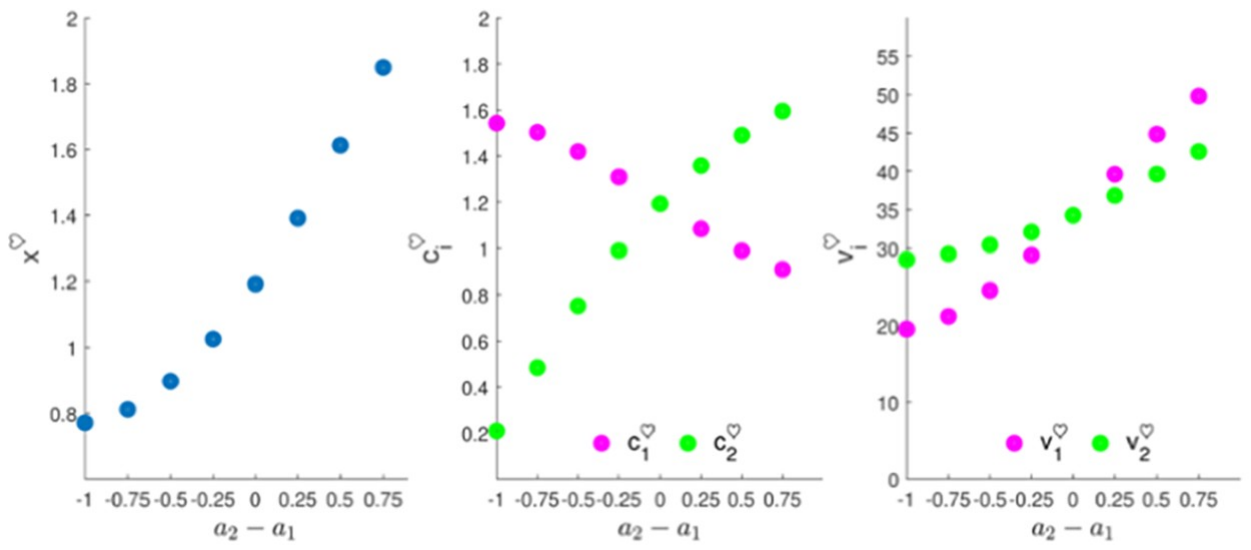
Long-term analysis of the 2D couple’s effort problem. The graphs show the equilibrium levels of feeling, and effort and total happiness of each partner versus the heterogamy gap *a*_2_ − *a*_1_ (with *a*_1_ = 1). Initial feeling is *x*(0) = 3.

Fig 4 displays the effort, feeling and well-being equilibrium levels versus the efficiency gap *a*_2_ − *a*_1_ (with *a*_1_ = 1) for the set of values of *a*_2_ used in the previous simulation. As explained above, the equilibrium feeling level increases as heterogamy (i.e. the efficiency gap) increases. As partner 2 becomes more efficient, his/her effort gap increases, whereas partner 1 benefits from that and his/her effort gap decreases. When it comes to happiness, both partners are better off when the efficiency gap increases. The partner whose effort is less efficient, however, obtains more happiness from the relationship.

**Fig 5:**
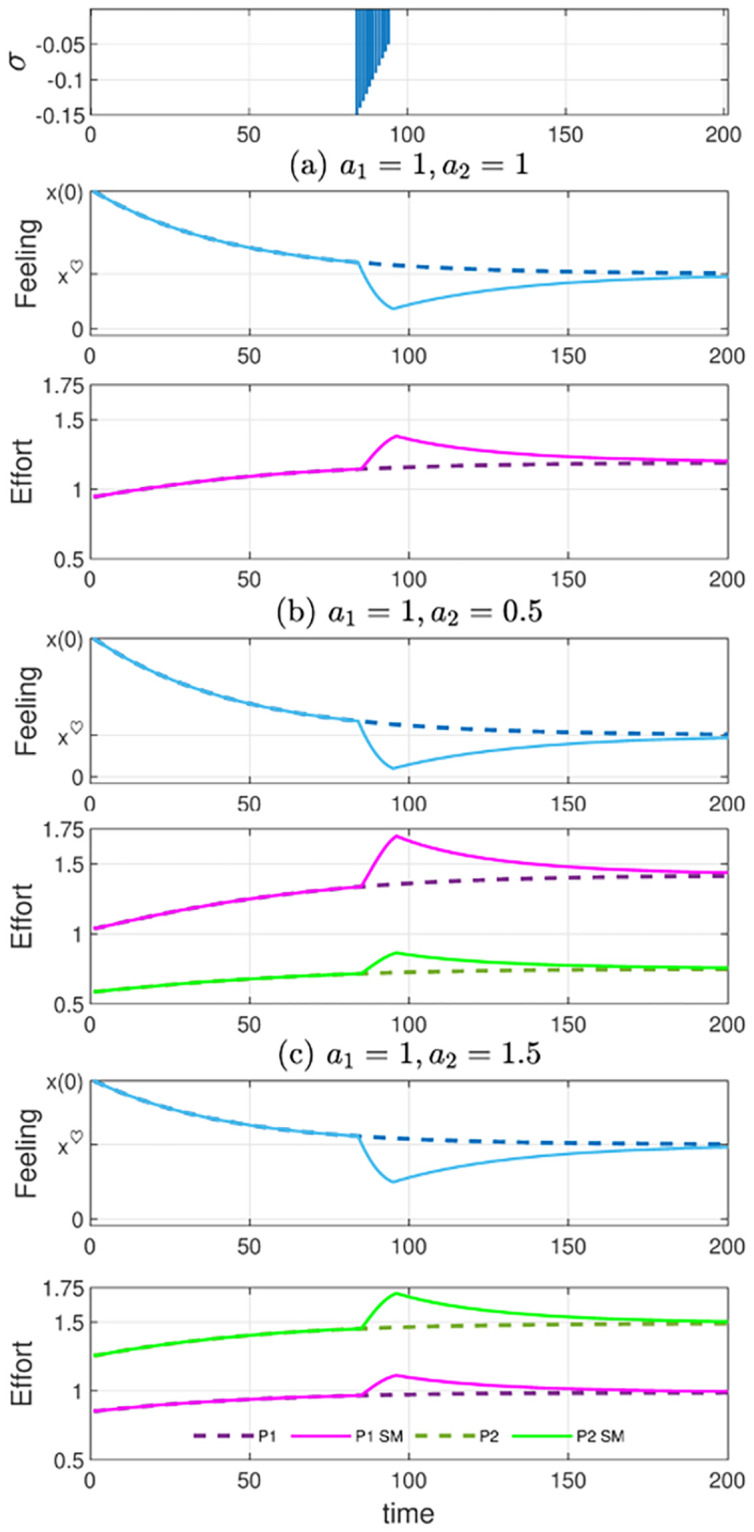
Feedback stabilization for the two-dimensional couple’s effort problem. The first panel shows the exogenous perturbation of the successful feeling trajectory. The sequence of shocks begins in the period *t* = 85 and takes values from −0.15 and −0.05, over 12 consecutive periods of time (same as Fig 2). The panels below show the corresponding feedback stabilizing solution. Panels (a), (b), and (c) show the corrected feeling trajectory and the stabilizing effort exerted by each partner for three different types of couples, i.e. (a) homogamous with *a*_1_ = 1 = *a*_2_, (b) heterogamous with *a*_1_ = 1, *a*_2_ = 0.5, and (c) heterogamous with *a*_1_ = 1, *a*_2_ = 1.5. In each panel, the discontinuous lines correspond to the unperturbed feeling and effort solutions. The stabilizing effort trajectories of partner 1 (with *a*_1_ = 1) are in purple in all panels. Initial feeling is *x*(0) = 3.

Fig 5 summarizes the analysis of the feedback stabilization mechanism for the 2D couple’s effort problem.

The first graph of the figure shows the sequence of exogenous shocks, which is the same as Fig 2. As explained there, the perturbation is consistent with a “seven-year itch”, assuming the month as the time period in our simulation. The stabilizing effort solution along with the corrected feeling trajectory for a homogamous couple with (*a*_1_ = *a*_2_ = 1) is compared with those of two heterogamous couples, namely with (*a*_1_ = 1, *a*_2_ = 0.5) and (*a*_1_ = 1, *a*_2_ = 1.5). The corresponding set of trajectories of each couple are plotted in panels (a), (b), (c). Feeling and effort trajectories follow a similar pattern for the different types of couples. During the stressful period, the feeling declines significantly deviating from the successful trajectory until reaching a nadir. Both partners must react promptly by increasing their effort to prevent the feeling from deteriorating further and take it back on the successful path. The successful path for each couple is plotted (with a dashed blue line), along with the destabilized trajectory (in solid blue), in the first graph of the corresponding panel.

The recovery process via feedback stabilization requires prolonged additional effort on the part of partners and it takes time. The stabilizing effort trajectories show that recovery time is affected by effort efficiency, that is, couples with higher aggregate efficiency recover their pre-shock feeling level earlier. This is further explored in the numerical experiment shown in Table 2. It analyzes the time required for different couples (with *a*_1_ = 1) initially at equilibrium to recover 95% of their pre-shock feeling level after a one-period perturbation of a given size. As *a*_2_ increases, the impact of the shock is lower relative to the feeling level, since this is greater as the aggregate efficiency increases. The most efficient couples, heterogamous or not, recover their pre-shock feeling earlier, as can be seen in the right column of the table.

**Table 2:** Analysis of the time recovery after a shock.

Parameters		% of the shock over *x*^♡^	time periods to recover 95% of *x*^♡^
*a*_1_	*a*_2_		
1	1.75	8%	≈ 20
	1.5	10%	24
	1	12.6%	36
	0.75	14%	≈ 48
	0.5	16%	≈ 60

For each type of heterogeneous couple at equilibrium, the right column shows the number of periods to recover the 95% of the level of feeling after a one-period shock of size −0.2. The middle column shows the relative size of the shock for each equilibrium feeling level.

Feeling and effort stabilizing trajectories in the dyadic case, shown in Fig 5, are qualitatively similar to those obtained for the 1D model (see Fig 2). The analysis of the dyadic problem, however, reveals significant effects due to heterogamy. First, panels (b) and (c) in Fig 5 show that the increase in effort during the critical period is more pronounced for the most efficient partner. Not only is his/her effort gap greater along the undisturbed period, but the additional effort required to deal with the shock is also greater. This asymmetry can be a serious source of discomfort for heterogamous couples since one partner can reach an unbearable level of effort. In principle, homogamous couples do not face such differences in their dedication to the relationship. They can probably coordinate and synchronize their matching contributions more easily.

The argument above may have implications for the gender gap in who wants to break up in heterosexual marriages. It is a well-known fact that women in the West are far more likely than men to want a divorce. This seems to be a unique feature of heterosexual marriages whose specific reasons remain unclear [24]. According to our simulation, if it were the case that wives are more efficient in their effort making, they would be more likely to experience severe stress in critical life episodes and, as a consequence, to want the breakup. It is uncertain whether the dedication and effort of wives in marriage have a greater impact on marital quality than those of men. However, if housework is somehow related to efficiency, there is evidence that wives who perceive their share of housework to be greater than that of their husbands are more likely to want a divorce [46]. Our results on efficiency disparity would therefore suggest a potential factor to explain the breakup gender gap.

## Conclusions

Long-term romantic relationships are fundamental social ties with an enormous impact on the well-being of individuals and societies. Still, how happy and stable relationships evolve is not well understood. This is a challenging problem for social scientists, in part due to the problem of obtaining noise-free quality data with homogeneous samples. More importantly, long-term longitudinal studies of married couples are scarce due to the difficulty of monitoring the same relationships for a long time.

In this article, feeling trajectories for synthetic couples are obtained by computer simulation, that can be prolonged for as long as necessary. The couples under study are initially very much in love, committed to building a common project and being happy together, and aware that this task requires deliberate effort. The effort problem of the couple amounts to find out the level of effort investment for their long-term project. This seems a central question in the field of relationship science.

The couple’s effort problem is formulated mathematically as a control problem, which the couple can solve jointly (as a single optimal control problem) or independently (as a differential game obeying a Nash rationale). A suitable algorithm is used to explore both versions of the problem. Regarding the effort pattern required to control the relationship successfully, the analysis provides evidence that effort making is demanding for the couple –relative to any favorite level a priori– and it must increase as time passes until it flattens out. In addition to that, the effort level must increase further under stressful periods in which the feeling is lowered by external influence.

Couples composed by similar individuals (homogamous) are known to be more stable. The synthetic couples considered here are essentially homogamous except for the impact their partners’ efforts have on the dynamics of the relationship.

Our analysis reveals that this slight heterogamy is relevant to well-being in the relationship. First, the stationary level of feeling depends not only on the amount of supplied effort but also on its quality, that is, efficiency. Secondly, individual efficiency matters: the most efficient partner has to make more effort.

Concerning the feeling dynamics, our analysis may contribute to the debate of how lifelong marital quality changes through time –see [10]. It is a consistent finding in the literature that marital quality initially declines for both successful and unsuccessful marriages, although the decline in happy marriages can be small. It is unclear how the relationship quality of successful couples evolves after that. There may not be a single successful pattern, as it has been suggested in the literature [47]. The goal is rather to identify conditions or traits of couples that are associated with different patterns of marital happiness. Our study suggests two different patterns for marital trajectories that are successful in the long run. Relationships that do not go through hard times or face stressful life events, like rearing children, exhibit a feeling curve showing a pattern of decay followed by a plateau stabilization.

These types of relationships do not experience any upward trend in happiness at a later time, as predicted by theoretical perspectives based on social psychological and individual processes.

On the other hand, successful couples who overcome stressful life events show a curvilinear feeling trajectory due to a relative increase in happiness in a later period. This pattern is observed in resilient relationships that implement a feedback stabilization mechanism.

Such U-shaped trajectories are consistent with the marital resilience perspective which relies on explanations based on changes in family roles and structures.

The computational analysis carried out in this article is complementary to field studies that collect real data. Carefully specifying the assumptions of the model allows us to control for particular traits of the synthetic couples under study. Since the assumptions of the computational model rely on preconditions obtained from studies of real couples, the synthetic trajectories obtained by simulation serve to approximate real-life trajectories that are not observable in the long term.

## Supporting information

S1 File (PDF)
