# Supplementary material for: Controlling forever love
Source: PLoS One. 2021 Dec 29;16(12):e0260529. doi: 10.1371/journal.pone.0260529 (PMC8716042; doi:10.1371/journal.pone.0260529)
Supplement: S1 File — (PDF) [file pone.0260529.s001.pdf]

Supporting information

Algorithm pseudocode

We present here the algorithm to compute the discretized feedback effort maps and value functions for the couple's effort problem. The goal is to find numerical solutions to the discretized HJB equations associated to the problem. The notation used below is the same as the main body of the article. First, we define a set of vectors and fix notations. Let V_i and C_i be the Q-dimensional arrays of values for the value functions, and controls for partner i evaluated at the set $\tilde{X}$:

$$\mathrm{V}_i = \left[\tilde{v}_i^h\left(y_1\right), ..., \tilde{v}_i^h\left(y_Q\right)\right]^T,\ \mathrm{C}_i = \left[\tilde{c}_i\left(y_1\right), ..., \tilde{c}_i\left(y_Q\right)\right]^T, i = 1, 2. \quad (1)$$

The notation $\mathrm{V} = [\mathrm{V}_1, \mathrm{V}_2]$, and $\mathrm{C} = [\mathrm{C}_1, \mathrm{C}_2]$, is used for the arrays storing the information for both partners.

Consider two fixed point operators $T_i = [T_{i,1}, ..., T_{i,Q}] : \mathbb{R}^Q \to \mathbb{R}^Q$ and $G_i = [G_{i,1}, ..., G_{i,Q}] : \mathbb{R}^Q \to \mathbb{R}^Q$ defined component-wise, for $j = 1, ..., Q$, by

$$T_{i,j}(\mathrm{V}_i) := h\left(U_i\left(y_j\right) - D_i\left(c_i\right)\right) + (1 - \rho_i h) RBF\left[\mathrm{V}_i\right]\left(y_j + hf\left(y_j, [c_1, c_2]\right)\right) \quad (2)$$

and

$$G_{i,j}(\mathrm{V}_i) := arg \max_{c_i \in \mathbb{R}^+} \left\{h\left(U_i\left(y_j\right) - D_i\left(c_i\right)\right) + (1 - \rho_i h) RBF\left[\mathrm{V}_i\right]\left(y_j + hf\left(y_j, [c_1, c_2]\right)\right)\right\}, \quad (3)$$

with $f(y, [c_1, c_2]) = -ry + a_1 c_1 + a_2 c_2$.

For $s = 0, 1, ...$ and $t = 0, 1, ...$ and define the following two-step process:

1. *Game Iteration.* First, generate a candidate C_i^{s+1} to optimal control policy at step $s + 1$ for both partners, as follows:

$$\mathrm{C}_i^{s+1} = \theta \mathrm{C}_i^s + (1 - \theta) G_i\left(\mathrm{C}^s, \mathrm{V}_i^r\right)\ i = 1, 2,$$

where G_i is defined in (3). The game iteration loop follows the scheme

$$\begin{cases} \tilde{c}_{1,j}^{s+1} \equiv \theta \tilde{c_{1,j}}^{s} + (1-\theta)\, arg\max_{c_1 \in \mathbb{R}^+} \left\{ h\left(U_1\left(y_j\right) - D_1\left(c_1\right)\right) + \left(1-\rho_1 h\right) RBF\left[V_1^r\right]\left(y_1^{\#}\right) \right\}, \\ \tilde{c}_{2,j}^{s+1} \equiv \theta \tilde{c_{2,j}}^{s} + (1-\theta)\, arg\max_{c_2 \in \mathbb{R}^+} \left\{ h\left(U_2\left(y_j\right) - D_2\left(c_2\right)\right) + \left(1-\rho_2 h\right) RBF\left[V_2^r\right]\left(y_2^{\#}\right) \right\}, \\ y_1^{\#} = y_j + hf\left(y_j, [c_1, \tilde{c_{2,j}}^{s}]\right), \\ y_2^{\#} = y_j + hf\left(y_j, [\tilde{c_{1,j}}^{s}, c_2]\right). \end{cases}$$

for $s = 0, 1, \ldots$, using $\tilde{c}_{i,j}^{s} \equiv \tilde{c}_i^{s}(y_j)$ to simplify notation. Here $0 < \theta < 1$ is a weighting coefficient-see [1]- and V_i^r are to be defined below. This scheme is iterated until a convergence criterion is satisfied, that is, $\left\|\mathrm{C}^{s+1} - \mathrm{C}^{s}\right\| < \epsilon_1$, for a given error $\epsilon_1 > 0$ (with $\|\cdot\|$ the Euclidean norm). A candidate for feedback Nash equilibrium, given the value functions V_i^r, is thus obtained

$$\mathrm{C}^{s+1} = \left[\mathrm{C}_1^{s+1}, \mathrm{C}_2^{s+1}\right].$$

This is an input of the next step, which aims to approximate the value functions V_i^{r+1}.

2. *Value iteration.* Once a candidate C^{s+1} for the feedback Nash equilibrium has been obtained from the previous game loop, the value functions at step $r+1$ are updated, as follows

$$\mathrm{V}_i^{r+1} = T_i\left(\mathrm{V}_i^r; \mathrm{C}^{s+1}\right),\ i = 1, 2,$$

where $T_i = [T_{i,j}]$ are defined component-wise, for $j = 1, ..., Q$, by the scheme

$$\begin{cases} T_{1,j} = h\left(U_1\left(y_j\right) - D_1\left(\tilde{c}_{1,j}^{s+1}\right)\right) + \left(1-\rho_1 h\right) RBF\left[V_1^r\right]\left(y_1^{\#}\right), \\ T_{2.j} = h\left(U_2\left(y_j\right) - D_2\left(\tilde{c}_{2,j}^{s+1}\right)\right) + \left(1-\rho_2 h\right) RBF\left[V_2^r\right]\left(y_2^{\#}\right), \\ y_1^{\#} = y_j + hf\left(y_j, \left[\tilde{c}_{1,j}^{s+1}, \tilde{c}_{2,j}^{s+1}\right]\right), \\ y_2^{\#} = y_j + hf\left(y_j, \left[\tilde{c}_{1,j}^{s+1}, \tilde{c}_{2,j}^{s+1}\right]\right), \end{cases}$$

using again the notation $\tilde{c}_{i,j}^{s} \equiv \tilde{c}_i^{s}(y_j)$. This second loop is iterated until satisfying $\left\|\mathrm{V}^{r+1} - \mathrm{V}^{r}\right\| < \epsilon_2$, with $\epsilon_2 > 0$ given, in turn obtaining a candidate for value

functions of the problem

$$V^{r+1} = \left[V_1^{r+1}, V_2^{r+1}\right].$$

Once the convergence criteria are reached after s and r iterations, respectively,

$$\mathrm{V}^r = [\mathrm{V}_1^r, \mathrm{V}_2^r],\ \mathrm{C}^s = [\mathrm{C}_1^s, \mathrm{C}_2^s]$$

are the computational outputs for the value functions and control policies of the problem. Notice that the numerical scheme above consists of a coupled system of the form $\mathrm{C}^{s+1} = G(\mathrm{C}^s, \mathrm{V}^r, \Delta)$ and $\mathrm{V}^{r+1} = T(\mathrm{V}^r, \mathrm{C}^{s+1}, \Delta)$, where Δ denotes spatio-temporal discretization parameters. The (approximate) fixed point $(\mathrm{C}^{\heartsuit}, \mathrm{V}^{\heartsuit})$ is the numerical solution for the couple's problem rendered by the algorithm for the chosen discretization Δ.

Radial basis functions.

The approximations $RBF[\mathrm{V_i}](\cdot)$, and $RBF[\mathrm{C_i}](\cdot)$, for $i = 1, 2$, are required by the numerical scheme to solve the HJB equations introduced in the main body of the article. They are also used to find the open-loop solutions defined by the iterative schemes (SM1) and (SM2) in the section Results and Discussion. They provide estimates of $\tilde{v}_i^h(y_i^\sharp)$ and $\tilde{S}_i^h(x)$, respectively, with $y_i^\sharp$ and x typically not in $\tilde{X}$, and are obtained using radial basis functions as follows:

$$\tilde{v}_i^h\left(y_i^{\#}\right) \approx RBF\left[\mathrm{V}_i\right] \equiv \sum_{j=1}^{Q} \lambda_{i,j} \Phi\left(\left\|y_i^{\#} - y_j\right\|\right),$$

$$\tilde{S}_i^h(x) \approx RBF\left[\mathrm{C}_i\right] \equiv \sum_{j=1}^{Q} \mu_{i,j} \Phi\left(\|x - y_j\|\right),$$

where $\lambda_{i,j}, \mu_{i,j} \in \mathbb{R}$, are weighting coefficients to be defined below, and $\Phi(\cdot)$ is a Gaussian real-valued radial basis function, i.e. $\Phi(\|y - y_j\|) = \exp\left(-\frac{\|y-y_j\|^2}{2\sigma^2}\right)$,with $\sigma > 0$ (see e.g. [2]). There is a number of different techniques to set the value of the parameter σ [3]. Following the procedure in [2], we have chosen $\sigma = 0.1$ as a suitable value.

For $i = 1, 2$, the weighting coefficients $\lambda_{i,j}, \mu_{i,j}, j = 1, ..., Q$, are obtained by solving

$$\begin{cases} \mathrm{A}\bar{\lambda_i} = \mathrm{V}_i \;\; \mathrm{A}\bar{\mu_i} = \mathrm{C}_i, \end{cases}$$

where A is the matrix with entries $\mathrm{A}_{jl} = \Phi\left(\|y_l - y_j\|\right)$, $j, l = 1, ..., Q$, $\bar{\lambda_i} = \left[\lambda_{i,1}, ..., \lambda_{i,Q}\right]^T$, $\bar{\mu_i} = \left[\mu_{i,1}, ..., \mu_{i,Q}\right]^T$, and $\mathrm{V}_i, \mathrm{C}_i$ are defined in (1).

Data availability for replications

The analysis of this article is carried out with synthetic data generated by a computational model, as explained in the main text of the article. The code has been written and run in MATLAB. The parameter values used in the computational experiments are: $h = 10^{-2}$, $\epsilon_1 = 10^{-3}$, $\epsilon_2 = 10^{-4}$, $Q = 15$, $x \in X = [0, 5]$, $\theta = 0.95$. The typical CPU time of the algorithm until convergence is around 2045s. A version of our code is available on the web page: http://www.mat.ucm.es/ ivorra/softrabvitg.htm. It can be used to replicate the main findings in this study. The necessary files are in the folder called *LOVE web*. All the necessary instructions are documented in the *MAIN* script.

References

1. Krawczyk JB, Uryasev S. Relaxation algorithms to find Nash equilibria with economic applications. Environmental Modeling & Assessment. 2000;5(1):63–73.

2. Fasshauer GE. Meshfree approximation methods with MATLAB. vol. 6. World Scientific; 2007.

3. Krowiak A, Podgórski J. On choosing a value of shape parameter in Radial Basis Function collocation methods. In: AIP Conference Proceedings. vol. 2116. AIP Publishing LLC; 2019. p. 450020.
